# Novel Iron-Chelating Prodrug Significantly Enhanced Fluorescence-Mediated Detection of Glioma Cells Experimentally In Vitro

**DOI:** 10.3390/pharmaceutics15122668

**Published:** 2023-11-24

**Authors:** Charlotte Reburn, George Gawthorpe, Alexis Perry, Mark Wood, Alison Curnow

**Affiliations:** Knowledge Spa, Royal Cornwall Hospital, University of Exeter, Truro TR1 3HD, UK; c.reburn@exeter.ac.uk (C.R.); gfg202@exeter.ac.uk (G.G.); a.perry@exeter.ac.uk (A.P.); m.e.wood@exeter.ac.uk (M.W.)

**Keywords:** fluorescence-guided resection (FGR), photodynamic therapy (PDT), iron chelation, protoporphyrin IX (PpIX), glioma, AP2-18, aminolaevulinic acid (ALA), MAL

## Abstract

(1) Background: The protoporphyrin IX (PpIX)-mediated fluorescence-guided resection and interoperative photodynamic therapy (PDT) of remaining cells may be effective adjuvants to the resection of glioma. Both processes may be enhanced by increasing intracellular PpIX concentrations, which can be achieved through iron chelation. AP2-18 is a novel combinational drug, which ester-links a PpIX precursor (aminolaevulinic acid; ALA) to an iron-chelating agent (CP94). (2) Methods: Human glioma U-87 MG cells were cultured in 96-well plates for 24 h and incubated for 3 or 6 h with various test compound combinations: ALA (±) CP94, methyl aminolevulinate (MAL) (±) CP94 and AP2-18. PpIX fluorescence was measured at 0, 3 or 6 h with a Bio-tek Synergy HT plate reader, as well as immediately after irradiation with a 635 nm red light (Aktilite CL16 LED array), representing the PDT procedure. Cell viability post-irradiation was assessed using the neutral red assay. (3) Results: AP2-18 significantly increased PpIX fluorescence compared to all other test compounds. All treatment protocols effectively achieved PDT-induced cytotoxicity, with no significant difference between test compound combinations. (4) Conclusions: AP2-18 has potential to improve the efficacy of fluorescence-guided resection either with or without the subsequent intraoperative PDT of glioma. Future work should feature a more complex in vitro model of the glioma microenvironment.

## 1. Introduction

### 1.1. Glioma and Fluorescence-Guided Resection

Glioma is a collective term for tumours originating from glial cells in the brain or spinal cord [1]. Current glioma treatment mortality rates are high, with most patients experiencing post-treatment tumour recurrence with poor prognosis (average survival after repeated resections is around 18 months) [2,3]. Around 75–80% of glioblastoma tumours recur locally, leading to increased risk of mortality in patients [4]. Treatment for glioma typically consists of surgical removal (resection), followed by chemotherapy and radiotherapy [5]. As well as having negative psychosocial impacts on patients, this conventional surgical resection method often leaves residual cancer cells behind after surgery, typically at tumour margins, which can lead to tumour recurrence [4,6]. 

Fluorescence-guided resection (FGR) can be utilised as an adjuvant to this conventional surgical method [7]. It involves detecting the naturally occurring fluorophore protoporphyrin IX (PpIX), which is elevated in cancer cells, following the administration of an oral PpIX prodrug precursor (a compound which can be metabolised within cells to become PpIX). This makes tumours fluoresce when exposed to activating light, typically of a wavelength of around 400 nm, and, thus, easier to detect and resect during surgery [7,8]. For example, seminal work conducted by Stummer et al. demonstrated a significant difference in glioma resection completeness when comparing those who underwent FGR to those who underwent resection under white light (65% vs. 36% complete resection, respectively) [7]. 

### 1.2. PpIX and the Haem Biosynthesis Pathway

**Figure 1:**
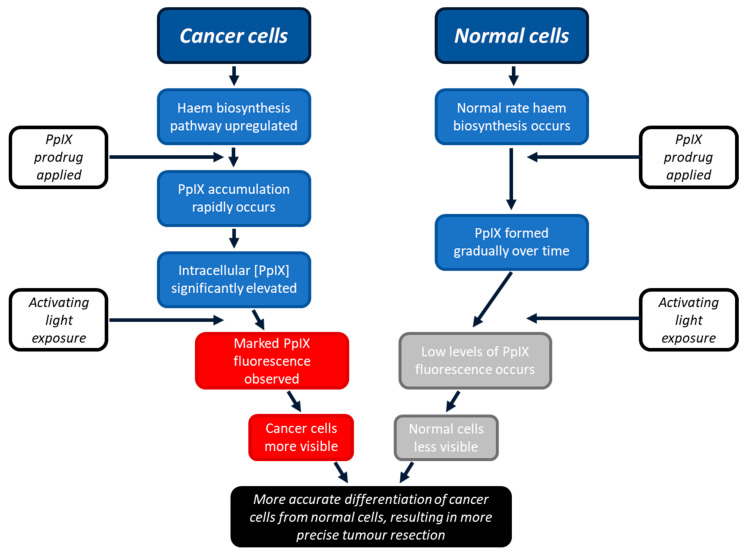
Schematic representation of the process through which PpIX can enhance FGR. Cancer cells become more fluorescent than surrounding normal cells as they have upregulated haem biosynthesis, resulting in greater temporary accumulation of PpIX. Healthy normal cells do not accumulate as much PpIX as quickly, as their innate haem biosynthetic pathway works at a slower, more controlled rate. This permits the two cell types to be differentiated, aiding more precise tumour detection and, thus, resection.

PpIX is produced by all nucleated cells during the innate haem biosynthetic pathway [9]. This pathway is upregulated in cancer cells [10]. PpIX is the immediate precursor of haem, and when an easily absorbed PpIX prodrug is applied to the lesion, either systemically or topically, higher levels of PpIX are formed because at the last step of the pathway, the insertion of iron into the porphyrin ring is rate limiting [10,11,12]. Examples of commonly used PpIX prodrugs include 5-aminolaevulinic acid (ALA) or ALA’s methyl ester, namely methyl aminolevulinate (MAL) [13]. Whilst the primary rate-limiting step is the formation of ALA, this is bypassed via exogenous PpIX prodrug administration, whereby the conversion of PpIX into haem becomes the rate-limiting step of importance. As a result, PpIX temporarily accumulates in the cell before its conversion into haem [10]. There is, therefore, an optimal window of time when there are high rates of PpIX accumulation in cancer cells and lower rates of PpIX accumulation in surrounding normal cells (within which period PpIX formation occurs in a more controlled manner). In the FGR of glioma, it is at this timepoint that the lesion should be alternately exposed to blue and white light to visualise the tumour PpIX fluorescence to aid more effective surgical resection (Figure 1) [13,14,15]. 

### 1.3. PpIX and Iron Chelation

In the haem biosynthesis pathway, Fe^2+^ ions bind to PpIX to form haem, catalysed by the enzyme ferrochelatase, reducing the amount of PpIX available to accumulate and naturally fluoresce [12]. Iron levels are, therefore, fundamental in intracellular PpIX build-up, and the manipulation of cellular iron concentration is pivotal in achieving maximum PpIX levels and fluorescence [12]. Iron chelators have been used experimentally to increase PpIX build-up in glioma [16,17], as have PpIX efflux inhibitors [8]. Iron addition has been shown to have a converse effect, decreasing intracellular PpIX [12]. 

Various iron chelators have been tested to determine how they affect the efficacy of PpIX build-up in different cell types, including skin and glioma cells [16,18,19]. The hydroxypyridinone iron chelator 1,2-diethyl-3-hydroxypyridine-4-one (CP94) is small, neutral, and lipophilic, and it can quickly enter cells and bond to intracellular iron, causing an increase in intracellular PpIX, as well as subsequent PpIX fluorescence [19]. It has been demonstrated that CP94 is more effective at increasing PpIX build-up compared to other iron chelators, such as deferoxamine (DFO) and ethylenediaminetetraacetic acid (EDTA) [19]. However, to our knowledge, it is yet to be evaluated in comparison to the new polymeric DFO iron-chelating agent that has recently been developed [20].

### 1.4. AP2-18, a Novel Prodrug

**Figure 2:**
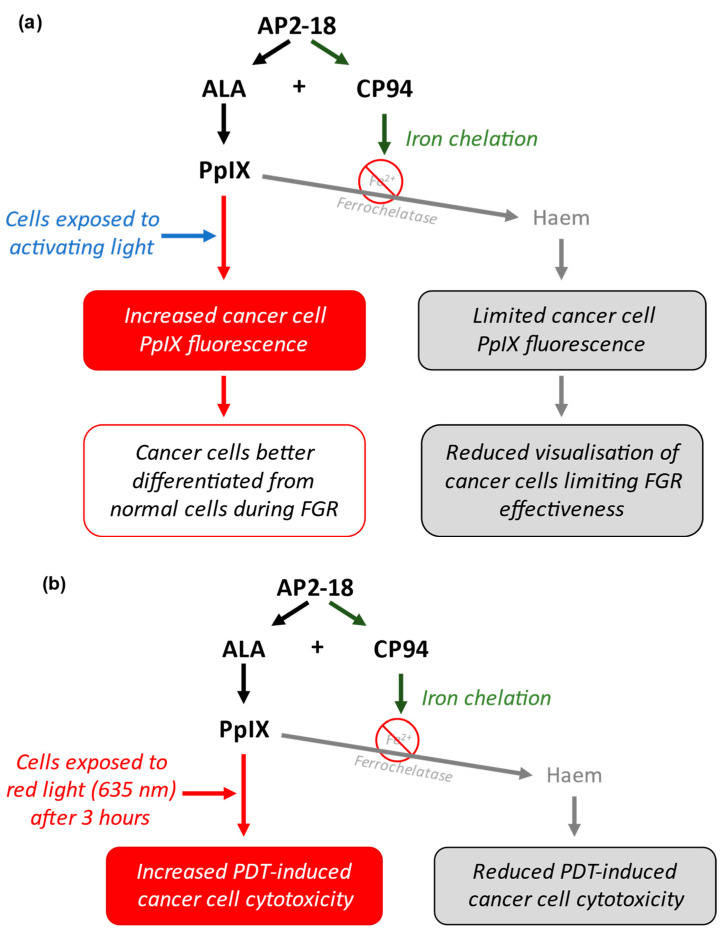
(**a**) Schematic representation of how AP2-18 could potentially enhance FGR. ALA and CP94 separate when inside cancer cells and can then act simultaneously. ALA acts as a PpIX prodrug, whilst CP94 chelates iron, reducing the iron-mediated conversion of PpIX into haem. This leads to increased intracellular [PpIX] and fluorescence when exposed to activating light (most commonly with a wavelength of around 400 nm), leading to enhanced visual differentiation between cancer and normal cells, aiding more effective surgical resection of the tumour. (**b**) Schematic representation of how AP2-18 could potentially enhance PDT. Increased [PpIX] induced via AP2-18-released CP94 iron chelation results in greater oxidative stress-induced cell damage of cancer cells upon 635 nm red light exposure, causing PDT-induced cell death.

The novel drug AP2-18 (synthesised by the University of Exeter) consists of the PpIX precursor ALA ester-linked to the iron chelator CP94 [Patent Application WO2014033477A1; 6 March 2014 [21]]. This highly soluble prodrug facilitates the simultaneous co-delivery of the two components in equimolar amounts. Previous studies conducted with AP2-18 have demonstrated its efficacy at enhancing PpIX build-up, with results suggesting that AP2-18 may be more effective at increasing PpIX build-up and fluorescence than the separate administration of similar concentrations of CP94 and either ALA or MAL. These studies took place in human lung fibroblasts, dermal fibroblasts, bladder cells and epithelial squamous cell carcinoma cells, and they led to increased PpIX fluorescence in all cell types apart from bladder cells [22,23]. These previous observations made in a range of different cell types indicate that there may be an opportunity to potentially enhance the FGR protocol for glioma, removing smaller lesions, or lesions in locations that are traditionally more difficult to treat due to difficulty in accessing them. This could provide improved post-surgery prognosis to patients with glioma by increasing the effectiveness of this procedure (Figure 2a).

### 1.5. Intraoperative Photodynamic Therapy in Glioma

Although increasing PpIX build-up and subsequent fluorescence would present an important improvement in FGR, it currently remains impossible to surgically resect all residual cancer cells around the tumour boundaries in what is sometimes referred to as the infiltrating zone. One technique that may further improve patient outcomes is intraoperative photodynamic therapy (PDT) [24]. PDT represents an important potential adjuvant to current surgical resection of glioma in patients, and it has been explored in many previous studies [25,26,27]. 

This process involves the administration of the same PpIX prodrugs (such as ALA and MAL, with or without an iron chelator) to lesions, followed by the irradiation of these lesions with a specific wavelength of red light (635 nm), typically 3 h after the administration of the prodrug [28]. ALA is normally administered to patients orally dissolved in a glucose solution for this particular application. A wavelength of 635 nm is used because this is a strong absorption peak of PpIX with good tissue penetration [29]. When simultaneously exposed to this light, as well as intracellular molecular oxygen, PpIX absorbs the energy from the light and transfers it to the molecular oxygen to initiate oxidative stress cascades [10,30]. When this occurs in sufficient amounts, overwhelming cellular damage results in the apoptosis or necrosis of the cell [23,30]. It has been demonstrated that PDT may be enhanced with the addition of hydroxypyridinone iron chelators such as CP94 [12]. Additionally, it has been hypothesised that using a combinational molecule such as AP2-18 could be more effective than utilising existing test compounds, accumulating more intracellular PpIX and having a greater cytotoxic effect on irradiation (Figure 2b) [22,23,31,32]. 

### 1.6. Aim of the Present Study

The aim of this study was to evaluate PpIX fluorescence levels and post-PDT cell viability in primary human glioma cells exposed to the novel drug AP2-18 compared to cells exposed to existing test compounds. These comparator compounds were ALA or MAL, with or without the addition of the iron chelator CP94. It was hypothesised that AP2-18 could increase PpIX fluorescence and post-PDT cell death in the glioma cells compared to the other drug combinations. PpIX fluorescence levels were measured as a proxy for an increase in FGR efficacy, whilst subsequent PDT-induced cell viability was monitored to assess the potential impact that this drug could have on increasing the cytotoxic effect of intraoperative PDT of glioma.

## 2. Materials and Methods

### 2.1. Cell Culture

All chemicals and reagents were purchased from Merck (Poole, UK) unless stated otherwise. Commercially available human glioma U-87 MG (ECACC 89081402) cells were purchased from the European Collection of Authenticated Cell Cultures (ECACC, Wiltshire, UK) and cultured in Eagle’s Minimum Essential Medium (EMEM), supplemented with 10% Foetal Bovine Serum (FBS), 2% Penicillin-Streptomycin (200 μL mL^−1^ and 200 μg mL^−1^ respectively) and 2% L-Glutamine solution (200 mM) by volume. Cells were incubated at 37 °C and 5% CO_2_ and passaged when necessary, typically every four to five days. All procedures were carried out inside a Category II laminar flow cabinet, in aseptic conditions. This cell type was selected based on previous studies of the efficacy of iron chelation on these cells [16].

### 2.2. Test Compound Preparation

Next, 100 mM stock solutions of the test compounds (ALA, MAL, CP94 and AP2-18) were prepared by diluting their powders in phosphate buffered saline (PBS, Thermo Fisher, Basingstoke, UK) and storing these solutions at 5 °C for up to one month. The solutions were diluted with colourless modified EMEM (with no phenol red, Thermo Fisher, Basingstoke, UK) on the day of each experiment to obtain the final concentrations of 250 μM and/or 500 μM. Colourless modified EMEM was utilised to avoid fluorescence measurement interference. A higher concentration of MAL was used than the concentration of ALA because MAL has been shown to be less effective than ALA in previous experiments, and these concentrations of the two compounds produced similar PpIX fluorescence and cell death results [19].

### 2.3. Incubation of Cells with Test Compounds

Cells were seeded in 96-well plates (Corning, Corning, NY, USA, flat bottom), at an average density of around 43,000 cells per well, 24 h prior to experimentation. Cells were incubated in the 96-well plates for 24 h in EMEM at 37 °C and 5% CO_2_. On the day of experiments, all media were removed from the wells, and all wells were washed twice with 100 μL of PBS, which was then removed. Next, 100 μL of test compound (ALA +/− CP94, MAL +/− CP94 or AP2-18) was added to each well. All procedures involving the contact of cells with test compounds were conducted in minimal light conditions to minimise premature PpIX excitation and prolong intracellular PpIX accumulation. There were 24 wells of cells in each plate, with 18 containing the 6 test compound combinations, which were 250 μM ALA +/− 250 μM CP94, 500 μM MAL +/− 500 μM CP94, and 250 μM and 500 μM AP2-18. Each test compound combination was applied to three wells. Six wells contained cells were incubated in colourless EMEM alone to act as controls. In each experiment, duplicate dark control plates were used alongside the light-exposed test plates. Dark control plates remained in darkness throughout the process, rather than being irradiated with red light (test plates). Experiments were replicated in triplicate on three separate days. 

### 2.4. PpIX Fluorescence Quantification

PpIX fluorescence was quantified using the Bio-tek Synergy HT plate reader (Bio-Tek Instruments Inc., Winooski, VT, USA), with an excitation wavelength of 405 ± 12 nm and an emission filter of 635 ± 12 nm. PpIX fluorescence measurements were taken immediately after test compounds were applied at 0 h, as well as at 3 or 6 h after incubation with the various test compound combinations and, finally, either immediately after irradiation or a period of darkness out of the incubator, for dark control plates. Between measurements, plates remained in the dark, incubated at 37 °C and 5% CO_2_. Before experimentation, PpIX fluorescence was aligned with concentration using a relative PpIX standard curve (in a manner similar to that we have performed previously [23]), where PpIX was dissolved to different concentrations, the fluorescence of which were measured using the plate reader. The PpIX emission spectra produced in cells from the various test compounds under investigation were also compared (in a manner similar to that we have performed previously [19]) to that of PpIX without note.

### 2.5. Photodynamic Therapy of Cells

PDT of the test plates occurred immediately after the PpIX fluorescence was measured at either the 3 or 6 h timepoint. Before treatment, all test compound solutions were removed from wells and replaced with 150 μL modified EMEM + 5% FBS to ensure that any subsequent damage caused by PDT was due to the PpIX that had already accumulated during the incubation time. A commercially available Aktilite CL16 LED array (Galderma, UK) was used to irradiate the cells (4 × 4 array head; 40 × 50 mm field size) at the more uniform irradiation distance of 8 cm with a narrow-band (full width at half maximum = 19 nm [33]), non-coherent red light at a dose of 37 J/cm^2^ and a wavelength of 635 ± 2 nm (75 mW/cm^2^). Temperature was not routinely monitored during this experimentation, as previous investigations had not highlighted this to be an issue. Dark control plates were removed from the incubator and kept in darkness for the same amount of time that the test plates were irradiated, which was 9 min, in accordance with the dose recommended in current UK NICE approved dermatological PDT guidelines [34]. 

### 2.6. Neutral Red Cell Viability Assay

The neutral red assay was used to measure cell viability after PDT treatment, 24 h after irradiation. Neutral red solution is taken up by the lysosomes in live cells through active transport. This process cannot occur in dead cells; hence, once the neutral red has been washed away, viable cells remain red, and dead cells are colourless [35]. Moreover, 45 min prior to this assay taking place, 15 μL of lysis solution (Triton X-100, 9% *v/v*, Promega, Madison, WI, USA) was added to three wells in each plate to act as a positive control for cell death. These three wells were only exposed to colourless EMEM prior to lysis. Plates were then incubated at 37 °C and 5% CO_2_ for 45 min. Meanwhile, the neutral red solution (10 mL) was centrifuged for 10 min at 1800 RPM to remove any crystals from the solution used. After 45 min incubation, the old colourless EMEM was gently removed from all wells, and 150 μL of fresh colourless EMEM was added, as well as 7.5 μL of neutral red solution. The plate was incubated for 1 h at 37 °C and 5% CO_2_, before the wells were washed with 150 μL PBS. This PBS was quickly removed and replaced with 150 μL of destaining solution (1% acetic acid in 50% ethanol solution) to dissolve the neutral red. This was left for 10 min, after which time the neutral red was released via gentle pipetting up and down. 

The Bio-tek Synergy HT plate reader was used to measure neutral red absorbance at a wavelength of 540 nm. Three wells in each plate, in which the cells had only been in contact with EMEM throughout, were used as baseline controls for cell viability. 

### 2.7. Data Analysis

Following the determination of parametric data sets, ANOVA, followed by post hoc *t*-tests, was used to determine the significance (at *p* < 0.05, 0.01, 0.001 and 0.0001) between the difference in the mean PpIX fluorescence values of the various test compound combinations at 3 or 6 h. Statistically significant differences in cell viability, as assessed using the neutral red assay, were also determined via ANOVA, followed by multiple paired, two-tailed *t*-test comparisons. Correlation analysis was conducted for the PpIX fluorescence and cell viability results for both timepoints (3 and 6 h). All data analysis was performed in GraphPad Prism 9. Results were presented as the mean PpIX fluorescence, with error bars showing the standard error of the mean (SEM) and mean cell viability according to the neutral red assay, with error bars showing standard deviation.

## 3. Results

### 3.1. PpIX Fluorescence 

**Figure 3:**
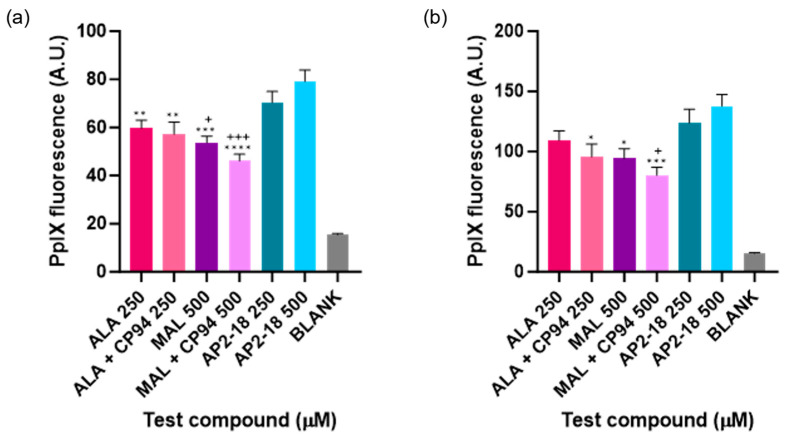
Mean PpIX fluorescence of human glioma cells (U-87 MG) after (**a**) 3 or (**b**) 6 h incubation of cells using various test compounds. The cells were incubated with ALA +/− CP94, MAL +/− CP94 or AP2-18 in various concentrations. CP94 was added in equimolar concentrations. Statistical significance (*t*-test) between AP2-18 and the other test compounds is indicated as * *p* < 0.05, ** *p* < 0.01, *** *p* < 0.001, **** *p* < 0.0001 or + *p* < 0.05, +++ *p* < 0.001. * corresponds to significance compared to AP2-18 at 500 μM, and + corresponds to significance compared to AP2-18 at 250 μM. Bars indicate the standard error of the mean (*n* = 3).

**Figure 4:**
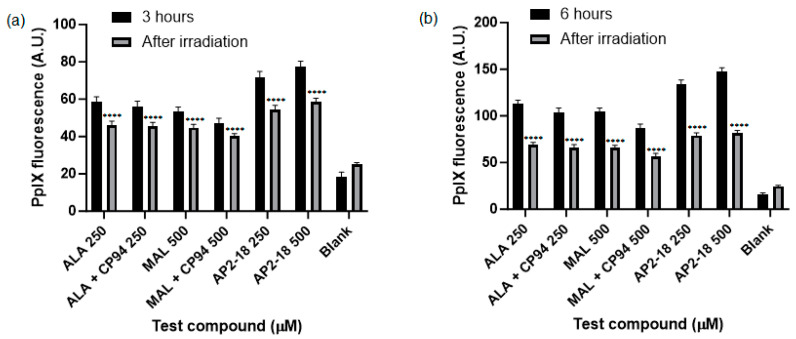
Mean PpIX fluorescence of human glioma cells (U-87 MG) was significantly decreased (*p* < 0.0001, *t*-test, as indicated by ****) immediately following irradiation (λ = 635 nm; light dose = 37 Jcm^−2^) after (**a**) 3 or (**b**) 6 h incubation of cells with all test compounds investigated. The cells were treated with ALA +/− CP94, MAL +/− CP94 or AP2-18 at various concentrations. CP94 was added in equimolar concentrations. Bars indicate the standard error of the mean (*n* = 3).

The level of PpIX fluorescence was quantified in each test group to determine which prodrug combination was the most effective at highlighting this human glioma U-87 MG cell type. A clear and significant increase in PpIX fluorescence (*p* < 0.05, ANOVA, followed by multiple *t*-test comparisons), resulted from treatment with AP2-18 at 250 and 500 μM at both 3 (Figure 3a) and 6 (Figure 3b) hours. The significant increase in PpIX fluorescence caused by AP2-18 was more pronounced at 3 h than at 6 h, with AP2-18 at 500 μM causing significantly higher levels of PpIX fluorescence than all the other test compounds at 3 h (*p* < 0.01, *t*-test), with the exception of AP2-18 at 250 μM (Figure 3a). Iron chelation with CP94 did not significantly alter PpIX fluorescence levels in the glioma cells at either 3 or 6 h (*p* > 0.05, *t*-tests). However, there was a visual trend towards iron chelation with CP94 resulting in reduced PpIX fluorescence, particularly when MAL was utilised as the PpIX precursor (Figure 3). As expected, irradiation with 635 nm light resulted in a significant decrease in PpIX fluorescence (*p* < 0.0001, *t*-test) for all test compounds at both time points (Figure 4), and this decrease was most pronounced in the cells treated with 250 or 500 μM AP2-18. 

### 3.2. PDT-Induced Cell Death

**Figure 5:**
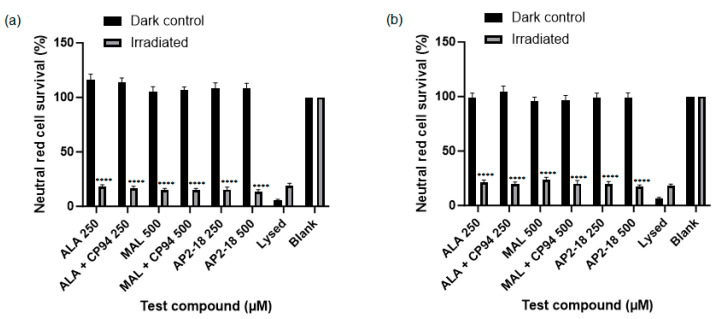
Mean percentage cell viability of human glioma cells (U-87 MG) according to the neutral red assay following PDT (λ = 635 nm; light dose = 37 Jcm^−2^) conducted at (**a**) 3 or (**b**) 6 h. Cells were treated with ALA +/− CP94, MAL +/− CP94 or AP2-18 in various concentrations. CP94 was added in equimolar concentrations. Statistical significance (*t*-test) between irradiated and dark control group cell viability for all test compounds was *p* < 0.0001, as indicated by ****, excluding lysed and blank controls for both time points. Bars indicate the standard error of the mean (*n* = 3).

**Figure 6:**
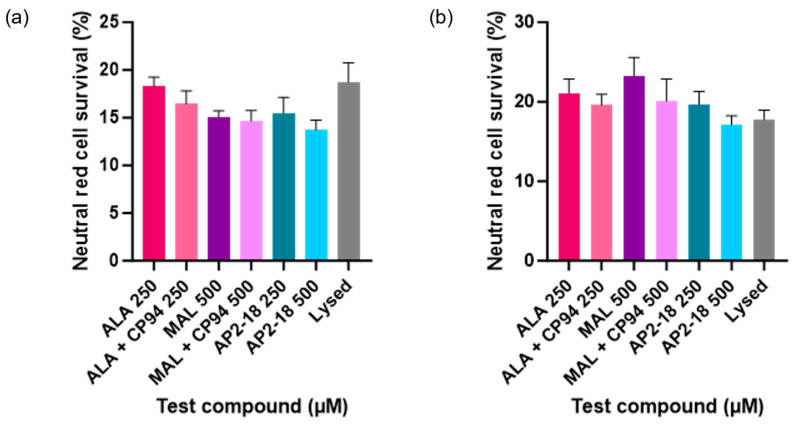
Mean percentage cell viability of human glioma cells (U-87 MG) following PDT conducted at (**a**) 3 or (**b**) 6 h (λ = 635 nm; light dose = 37 Jcm^−2^) according to the neutral red assay. Cells were treated with ALA +/− CP94, MAL +/− CP94 or AP2-18 in various concentrations. CP94 was added in equimolar concentrations. All differences between test compounds were non-significant (ANOVA). Bars indicate the standard error of the mean (*n* = 3).

Human glioma (U-87 MG) cell viability, as assessed using the neutral red assay, was ascertained following PDT irradiation to investigate which prodrug combination was the most effective at killing this cancer cell type. Statistically significant cytotoxicity (*p* < 0.0001, *t*-test) was observed in all PDT irradiated cells incubated with the test compounds, compared to the corresponding dark controls at both 3 (Figure 5a) and 6 h (Figure 5b). No significant dark toxicity was observed for any of the test compounds, with cell viability levels according to the neutral red assay being similar to those of the control cells, which were incubated in cell culture media (EMEM) alone (Figure 5). All PDT test compound combinations, including the novel combinational AP2-18 prodrug, were highly effective at killing the cultured glioma cells, resulting in a decrease in cell viability of at least 70% upon irradiation. There was a visual trend of AP2-18 causing slightly increased PDT-induced cytotoxicity compared to other test compounds (Figure 6), but this did not reach statistical significance (*p* > 0.05, ANOVA, followed by multiple *t*-test comparisons). 

**Figure 7:**
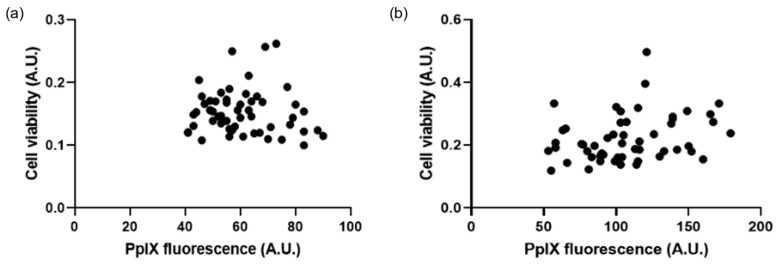
Correlation graphs show the lack of relationship between PpIX fluorescence pre-PDT and cell viability post-PDT. At (**a**) 3 h, R-squared value = 0.08 (*n* = 3), and (**b**) 6 h, R-squared value = −0.07 (*n* = 3).

Additional analysis was then subsequently conducted to consider any potential relationship between PpIX fluorescence levels pre-PDT and cell viability post-PDT. This correlation analysis was determined to not be significant (*p* > 0.05) (Figure 7).

## 4. Discussion

### 4.1. PpIX Fluorescence 

#### 4.1.1. AP2-18 Significantly Increased PpIX Fluorescence

AP2-18 significantly increased PpIX fluorescence in U-87 MG glioma cells compared to the administration of ALA or MAL, with or without the separate administration of CP94 (Figure 3). The increase in PpIX fluorescence as a result of AP2-18 has been observed in previous studies in various cell types [22,23]. There are several possible explanations for this observation. Firstly, with AP2-18, the PpIX prodrug ALA and the iron chelator molecule CP94 not only enter cells at exactly the same time, but they also do so in equal concentrations, which, in the case of glioma cells, may have resulted in intracellular concentrations of the two molecules being closer to the optimal ratios needed for most effective PpIX production [23]. Zhou et al.’s work [31] also suggests that an ester linkage between the ALA PpIX precursor and the iron-chelating agent would increase the lipophilicity of the combined molecule [32]. This would enable the molecule to cross the cell membrane more readily, entering the cells through simple diffusion [32], leading to increased PpIX accumulation compared to the separate administration of the PpIX prodrug and iron-chelating molecules. Furthermore, the iron-chelating agent employed within the combined structure of AP2-18, CP94, has a lower molecular mass and neutral charge and greater lipophilicity compared to other types of iron-chelating agent such as DFO or EDTA. These properties may, therefore, have also contributed to the significantly elevated PpIX levels detected here as a result of AP2-18 incubation with glioma cells.

The experimental increase in PpIX fluorescence caused by AP2-18 has important potential clinical applications. The increased fluorescence of tumour areas during the FGR process could help to discern healthy tissue from any areas of neoplastic tissue more clearly, which could lead to improvements in the accuracy of the FGR process [36] and may, with appropriate research, lead to improved outcomes for cancer patients in the future. Improving the accuracy of glioma FGR is also of vital importance, as any resection of healthy brain tissue could be life altering. 

#### 4.1.2. AP2-18 Increased PpIX Fluorescence in a Shorter Time Frame than Other Compounds

Another potential clinical advantage of using AP2-18 for FGR (rather than the traditional photosensitising agents, with or without the separate administration of an iron chelator), could be the more rapid accumulation of PpIX fluorescence levels observed in the glioma cells. Figure 3 indicates that the highest concentration of AP2-18 investigated here (500 μM) resulted in nearly as much PpIX fluorescence after 3 h as those of some of the other separate test combinations achieved after incubation with the glioma cells for twice as long (6 h). As performing FGR within the brain is a major surgical procedure, any factor that could potentially achieve adequate PpIX fluorescence levels in a shorter time frame represents important progress. 

#### 4.1.3. Separate Administration of CP94 Appeared to Decrease PpIX Fluorescence

Although this visual trend did not reach statistical significance, it appeared that the administration of CP94 alongside both ALA and MAL caused less PpIX to accumulate in the cells, and, thus, less PpIX fluorescence, compared to administration of either prodrug without CP94. This was not anticipated, as previous studies by this group have demonstrated that CP94 addition results in a significant increase in PpIX fluorescence in other cell types [12,19], including glioma [16]. Without a detailed molecular analysis of these intracellular processes, it is difficult to ascertain exactly why this trend appeared in the present study. However, it is known that CP94 enters cells via simple diffusion, whilst ALA enters cells through different modalities, including GABA receptors, depending on the cell type, which may mean that CP94 enters the cells more readily than ALA or MAL [23,37,38]. The separate administration of these molecules may, therefore, have led to CP94 entering the glioma cells faster, and this may have reduced the amount of available intracellular iron to levels that altered cellular functions such as respiration to be compromised. In the previous studies of the CP94 enhancement of PpIX-induced PDT, including experiments in U-87 MG cells [16], the CP94 concentrations that were employed were substantially lower than equimolar ones utilised here, so it is difficult to draw firm conclusions on the reasons for this non-significant observation without further detailed experimentation, as we know how sensitive this complicated biochemical cellular system is to free iron levels [12].

### 4.2. PDT-Induced Cell Death

#### 4.2.1. PDT-Induced Cell Death Was Achieved with All Test Compound Combinations

AP2-18 was found to be an effective photosensitiser for PDT-induced cytotoxicity in the glioma cells studied (Figure 5 and Figure 6). This concurs with previous work conducted with this compound, where it was found that PDT with AP2-18 caused high levels of cell death in both squamous cell carcinoma cells and lung fibroblasts [22]. A significant decrease in PpIX fluorescence, or photobleaching, post-PDT was detected (Figure 4). This photobleaching represented a very small portion of PpIX itself being converted into a (non-PDT active) photoproduct, as the PDT reactions proceeded via oxidative reactive oxygen species (ROS) cascades, producing the cytotoxic effects observed here [10]. This implies that as well as being a tool for the efficient FGR of glioma, AP2-18 could be an effective photosensitiser for use for intraoperative PDT, potentially destroying residual neoplastic cells at tumour margins, as it has previously been documented that PpIX photobleaching is correlated with PDT effectiveness [11]. 

#### 4.2.2. Low Cytotoxicity Observed in Control Cells

Importantly, low levels of cytotoxicity were observed in the control groups (Figure 5). Cell viability amongst dark control groups remained the same as the media-only control groups that were not exposed to any test compounds. Similarly, the light control groups that were incubated with cell culture media and subsequently irradiated also experienced little cytotoxicity. In combination, these findings re-illustrate, via this test system, the well-known fact that in order for PDT to achieve cytotoxicity, both the photosensitiser and activating wavelength of light must be simultaneously present within cells in sufficient quantities (concurrently with molecular oxygen) [10]. These findings are of great clinical relevance because they indicate that when utilising this approach in patients, healthy tissue surrounding the tumour(s) being treated should experience little or no cytotoxicity. The minimisation of any undesirable collateral damage to healthy cells caused by the treatment, particularly when treating glioma in the brain, is imperative and a major advantage of this developing treatment modality.

#### 4.2.3. Lack of PpIX Fluorescence Correlation with Post-PDT Cell Viability

Although AP2-18 increased PpIX fluorescence significantly and achieved significant cell kill upon PDT, the reduction in cell viability post-PDT did not correlate to the amount of PpIX fluorescence detected pre-PDT for any test compound (Figure 7). This was expected as PDT in vitro is highly effective with the established PpIX-precursors, namely ALA and MAL, and all the concentrations of the test compounds utilised here were selected on the basis of their previous effectiveness levels [19]. Despite stark differences in the PpIX fluorescence levels for the different test compounds being observed at both 3 h and 6 h (Figure 3), this did not result in any significant changes in cytotoxicity (Figure 6), indicating that the PpIX fluorescence levels produced in all the test groups investigated were cytotoxically effective in this U-87 MG cell type (i.e., exceeded the oxidative damage threshold to induce cell death, noting that this outcome is a dichotomous event, not a gradual process that can be continuously improved). The new AP2-18 combinational prodrug has, therefore, been demonstrated to be just as effective at causing PDT cytotoxicity as the other established PpIX-precursors but with the major advantage of significantly increasing PpIX fluorescence that could potentially harnessed to improve the FGR of glioma either with or without subsequent adjuvant PDT treatment. 

### 4.3. Limitations of the Present Study and Areas for Future Research

Glioma consists of a complex mass of different cell types, and whilst the cell model employed in this study was an effective tool for initial experimentation with the novel iron-chelating PpIX-prodrug AP2-18, as with every model, it was highly simplified in comparison to the complex system that it represented [39,40]. For example, the way the drugs interact with the blood–brain barrier, and its permeability may have an impact on PpIX accumulation in practice [41]. PDT effectiveness may also depend upon the cellular environment in which it is acting. When a tumour undergoes surgical resection, the concentration of the immunomodulatory molecule IL-6 increases around the areas of trauma [42], and this increase in inflammation may lead to an increase in blood pressure and local bleeding [43,44]. All these factors may affect the efficacy of in vivo intraoperative PDT. Therefore, evaluating AP2-18 in a more complex in vitro model in the future, which could better represent the complexities of the glioma microenvironment, would be desirable. 

Future work should also include flow cytometry experimentation to both confirm the neutral red cell viability results observed here and further elucidate the manner of cell death (apoptosis and/or necrosis) being induced using the different PDT treatment parameters. Moving forward, it would also be of great interest to consider intracellular ROS generation [30] and cellular damage [23] in much greater depth than we have previously in this emerging application of great clinical interest.

The specifics of the PDT procedure utilised here, including light dose, have been adapted for in vitro experimentation from those employed for licensed dermatological applications [28,34]. Studies of PDT treatment of glioma have utilised doses of up to 200 Jcm^−2^ [25] with durations up to 60 min [26]. As this emerging field continues to develop, further consideration should be given to the parameters adopted for corresponding in vitro investigation. Special attention should be paid to the results of the INDYGO clinical trial [45], which is currently investigating the efficacy of PDT conducted after FGR to try to improve glioma prognosis with very promising initial results indicating that the procedure is safe and reduces tumour reoccurrence [25].

## 5. Conclusions

The novel iron-chelating PpIX-prodrug AP2-18 has been demonstrated to significantly increase PpIX fluorescence accumulation in U-87 MG human glioma cells in vitro. An experimental finding, which with appropriate further study may have the potential to improve FGR clinical detection and, thus, surgical removal of glioma in the future, was identified. AP2-18 has also been demonstrated to act as an effective photosensitising agent for PDT, achieving high levels of cytotoxicity with activating light exposure in this test system. Although these experimental results do not reflect complete tumour clearance in vivo, this study has demonstrated the potential for improvement if these techniques were utilised concurrently to treat glioma in patients, and as a result, they warrant further detailed investigation. The utilisation of intraoperative PDT as an adjuvant therapy alongside the FGR of the visible tumour could kill residual cancer cells remaining within the infiltrating zone at the tumour margins, while sparing healthy cells from unnecessary damage. This could potentially convey substantial survival benefits to patients with glioma, as these are usually the cells that cause tumour regrowth to recur. 

## Data Availability

The data can be shared on request.
